# Retrospective validation of automatic sleep analysis with grey areas model for human‐in‐the‐loop scoring approach

**DOI:** 10.1111/jsr.14362

**Published:** 2024-10-23

**Authors:** Matias Rusanen, Gabriel Jouan, Riku Huttunen, Sami Nikkonen, Sigríður Sigurðardóttir, Juha Töyräs, Brett Duce, Sami Myllymaa, Erna Sif Arnardottir, Timo Leppänen, Anna Sigridur Islind, Samu Kainulainen, Henri Korkalainen

**Affiliations:** ^1^ Department of Technical Physics University of Eastern Finland Kuopio Finland; ^2^ Diagnostic Imaging Centre Kuopio University Hospital Kuopio Finland; ^3^ HP2 Laboratory, INSERM U1300 Grenoble Alpes University Grenoble Alpes University Hospital Grenoble France; ^4^ Reykjavik University Sleep Institute, School of Technology Reykjavik University Reykjavik Iceland; ^5^ Science Service Centre Kuopio University Hospital Kuopio Finland; ^6^ School of Electrical Engineering and Computer Science The University of Queensland Brisbane Queensland Australia; ^7^ Princess Alexandra Hospital Sleep Disorders Centre, Princess Alexandra Hospital Brisbane Queensland Australia; ^8^ Landspitali–The National University Hospital of Iceland Reykjavik Iceland; ^9^ Department of Computer Science Reykjavik University Reykjavik Iceland

**Keywords:** Convolutional neural networks, Human‐in‐the‐loop, Sleep staging, Uncertainty mapping

## Abstract

State‐of‐the‐art automatic sleep staging methods have demonstrated comparable reliability and superior time efficiency to manual sleep staging. However, fully automatic black‐box solutions are difficult to adapt into clinical workflow due to the lack of transparency in decision‐making processes. Transparency would be crucial for interaction between automatic methods and the work of sleep experts, i.e., in human‐in‐the‐loop applications. To address these challenges, we propose an automatic sleep staging model (aSAGA) that effectively utilises both electroencephalography and electro‐oculography channels while incorporating transparency of uncertainty in the decision‐making process. We validated the model through extensive retrospective testing using a range of datasets, including open‐access, clinical, and research‐driven sources. Our channel‐wise ensemble model, trained on both electroencephalography and electro‐oculography signals, demonstrated robustness and the ability to generalise across various types of sleep recordings, including novel self‐applied home polysomnography. Additionally, we compared model uncertainty with human uncertainty in sleep staging and studied various uncertainty mapping metrics to identify ambiguous regions, or “grey areas”, that may require manual re‐evaluation. The validation of this grey area concept revealed its potential to enhance sleep staging accuracy and to highlight regions in the recordings where sleep experts may struggle to reach a consensus. In conclusion, this study provides a technical basis and understanding of automatic sleep staging uncertainty. Our approach has the potential to improve the integration of automatic sleep staging into clinical practice; however, further studies are needed to test the model prospectively in real‐world clinical settings and human‐in‐the‐loop scoring applications.

## 1 INTRODUCTION

Manual sleep staging constitutes a fundamental analysis conducted in sleep clinics worldwide (Carskadon and Dement, 2011). This process involves the examination of specific patterns in electroencephalography (EEG), electro‐oculography (EOG), and chin electromyography (chin EMG) within 30‐second segments i.e., epochs of sleep recordings. Each epoch is subsequently categorised into one of five sleep stages: Wake, non‐rapid eye movement sleep stages 1–3 (N1, N2, or N3), or rapid eye movement (REM) sleep. The established scoring rules are applied universally to guide this task (Berry et al., 2020). Despite the diligent efforts invested in manual sleep staging, the presence of ambiguous regions persists, characterised by instances in which the expert scorers are unable to reach a consensus and unintentionally introduce errors (Lee et al., 2021; Van Gorp et al., 2022; Zhang et al., 2015).

Uncertain areas with discrepancies between scorers, i.e. grey areas, of sleep staging exist for multiple reasons. Firstly, the scoring rules employed for sleep staging have faced criticism for insufficient scientific foundation and the potential for interpretive variations (Himanen & Hasan, 2000; Jorgensen et al., 2020; Parrino et al., 2009). Additionally, scoring practices differ between sleep centres, as each institution usually adopts its own set of national or internal practices in addition to the traditionally used American Academy of Sleep Medicine scoring manual (Arnardottir et al., 2016; Norman et al., 2000). Moreover, the simplification of scoring sleep stages into 30‐second epochs can lead to confusion, particularly during transitions between stages (Schulz, 2008). Furthermore, sleep recordings are susceptible to artefacts, which can impact the accuracy of sleep stage analysis. In some instances, these artefacts may mimic characteristic patterns observed in EEG, EOG, or EMG traces specific to sleep stages (Kalevo et al., 2019). While skilled sleep technologists can perform sleep staging relatively accurately, even under such challenging circumstances (Nikkonen et al., 2023), the manual sleep staging necessitates substantial effort and an extensive allocation of work hours (Fischer et al., 2012).

To alleviate the challenges associated with manual sleep staging, numerous automatic sleep staging solutions have been devised (Fiorillo et al., 2019; Phan & Mikkelsen, 2022). These solutions have already been integrated into diverse polysomnographic (PSG) analysis software and are sometimes employed for preliminary scoring, which may speed up and increase the reliability of the manual sleep staging process (Id & Rijsman, 2022). Machine learning (ML)‐based methods, representing the cutting‐edge in this domain, demonstrate comparable performance to expert scorers in sleep staging, particularly in specific datasets on which the models were trained (Alvarez‐Estevez & Rijsman, 2021; Fiorillo et al., 2019; Fiorillo et al., 2021; Huttunen et al., 2022; Olesen et al., 2021; Perslev et al., 2021; Phan & Mikkelsen, 2022; Phan, Mikkelsen, et al., 2021; Stephansen et al., 2018). Further studies are needed to test the generalisability of the methods across different datasets and recordings conducted on various medical sensor setups. Moreover, based on current clinical recommendations, automatic sleep analysis tools cannot be relied upon solely, and their results necessitate thorough manual review (Goldstein et al., 2020). Only a few solutions have been introduced for highlighting the uncertain regions of automatic sleep analysis (Bakker et al., 2022; Fiorillo et al., 2021; Hong et al., 2021; Kang et al., 2021; Phan, Mikkelsen, et al., 2021), and the concept warrants further validation. In particular, it remains unclear whether these uncertain regions correspond to areas where confusions between manual scorers emerge, and therefore, a careful manual review of these areas could increase the quality of sleep staging. Moreover, conceptualisation and discussion regarding the interaction between automatic and manual scoring remains limited (Goldstein et al., 2020; Van Gorp et al., 2022). This discussion would be crucial for the consideration of human‐in‐the‐loop approaches in sleep analysis where humans play a crucial role in guiding and validating the ML system's decisions, ensuring that they are clinically reasonable and aligned with human based scoring (Monarch, 2021).

**FIGURE 1:**
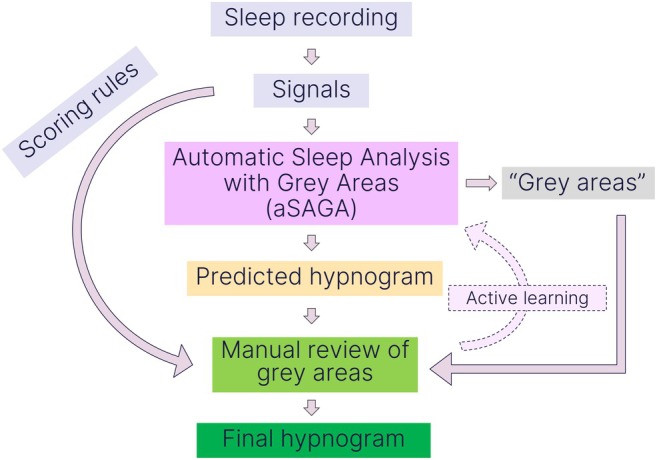
The proposed human‐in‐the‐loop sleep staging concept for the interaction of manual and automatic methods. In this approach, the aSAGA model gives a prediction of the hypnogram and highlights uncertain regions, the grey areas, that need manual re‐evaluation. In this paper, we aimed to evaluate the aSAGA model with retrospective data. In future work, active learning can be incorporated as a feedback loop to the proposed approach.

This paper presents a novel conceptualisation of the interaction between manual and automatic sleep staging (Figure 1), where we propose a model called aSAGA (Automatic Sleep Analysis with Grey Areas) to realise the human‐in‐the‐loop concept. This model integrates a state‐of‐the‐art automatic sleep staging method that exhibits generalisability across different recording types (Rusanen et al., 2023), along with transparency of decision‐making through uncertainty mapping. The uncertainty mapping is based on the hypodensities (Anderer et al., 2023; Stephansen et al., 2018), representing the probabilities of being in each sleep stage for each epoch. We hypothesise that the hypnodensities can be used to highlight areas of the recording where detailed manual revision can lead to an increased quality of the sleep staging analysis. The aim is to assess whether the grey areas identified by the model correspond to regions where discrepancies arise between automatic and manual scoring as well as between sleep technologists. Furthermore, we aim to compare the effectiveness of hypnodensity‐based uncertainty metrics introduced in the literature and to discuss the possible strategies to define the grey areas.

## 2 METHODS

### 2.1 Datasets

#### 2.1.1 Clinical PSG recordings

The clinical in‐laboratory PSG recordings used in the training of the automatic sleep staging model are described in detail in a previous work (Huttunen et al., 2022). These recordings form a heterogeneous clinical dataset of suspected obstructive sleep apnea (OSA) patients collected at the Princess Alexandra Hospital (Brisbane, Australia) during the years 2015–2017 (Table S1–S3). These recordings were conducted at the hospital's sleep laboratory with Compumedic (Abbotsford, Australia) Grael 4K PSG system with practices in line with the AASM guidelines (Berry et al., 2020). The Institutional Human Research Ethics Committee of the Princess Alexandra Hospital reviewed and approved a research proposal for the use of these data (HREC/16/QPAH/021 and LNR/2019/QMS/54313). We excluded recordings with less than 1 h of total sleep time and divided the data randomly into training (*n* = 710), validation (*n* = 78), and test sets (*n* = 88). Demographic information of the data is presented in Data **S1**.

#### 2.1.2 Dreem open data

The Dreem Open Data (DOD) is a documented and freely available open‐access PSG dataset (Guillot et al., 2020). The data consist of 25 healthy participants (DOD‐H) and 56 participants with OSA (DOD‐O). The DOD‐H data were collected at the French Armed Forces Biomedical Research Institute's (IRBA) Fatigue and Vigilance Unit using Siesta PSG devices (Compumedics, Victoria, Australia). The data included 13 EEG derivations (C3‐M2, F4‐M1, F3‐F4, F3‐M2, F4‐M2, F4‐O2, F3‐O1, FP1‐F3, FP1‐M2, FP1‐O1, FP2‐F4, FP2‐M1, and FP2‐O2) as well as derivations for right and left EOG. The DOD‐O data were collected at the Stanford Sleep Medicine Centre using a Somno HD PSG device (Somnomedics). These data included eight EEG derivations (C3‐M2, C4‐M1, F3‐F4, F3‐M2, F4‐O2, F3‐O1, FP1‐M2, and FP1‐F3) along with the left and right EOG. In both datasets, each record was scored by five expert sleep technologists (>5 years of clinical/scoring experience) from three different sleep centres. Further details on DOD data can be found in the original article (Guillot et al., 2020). The DOD data were used as a separate test set in this study, i.e., not in the training of the model. An end‐to‐end example of how to use these data for training an automatic sleep staging model is available at https://github.com/UEF-SmartSleepLab/sleeplab-format/tree/main/examples/dod_sleep_staging.

#### 2.1.3 Self‐applied PSG data

The self‐applied PSG recordings were collected at Akershus University Hospital in Norway and the Reykjavik University Sleep Institute in Iceland as a part of the Sleep Revolution Horizon 2020 project (ethical permissions 21–070 (Iceland) and 2017/2161 (Norway)) (Arnardottir et al., 2022; Sveinbjarnarson et al., 2023). The goal was to get a balanced cohort of healthy subjects and different levels of sleep apnea severity. A total of 50 participants received the self‐applied PSG (Self‐Applied Somnography (SAS), Nox Medical, Reykjavik, Iceland) equipment to take home for one night, along with written and video instructions on the setup. The SAS data comprised four EEG (AF4‐E3E4, AF3‐E3E4, AF7‐E3E4, and AF8‐E3E4) derivations and four EOG (E1‐E4, E2‐E3, E2‐AFZ, and E3‐AFZ) derivations that were used in the analysis. Demographic information of the data is presented in the supplementary material (Table S4).

Ten expert sleep technologists from seven different sleep centres scored each of the 50 recordings. Each scorer used Noxturnal Research version 6.1.0 (Nox Medical, Reykjavik, Iceland) software to first run an integrated automatic analysis and then inspected and scored/corrected the scoring following the AASM 2.6 scoring manual (Berry et al., 2018). This scoring protocol was used to correspond to common clinical practice. In the end, we obtained 10 scorings for each recording. A majority score for sleep stages was generated by selecting the most‐scored sleep stage for each epoch for the 10 scorers (Nikkonen et al., 2023). A tiebreaker method was used to choose the sleep stage with higher priority in the following order: Wake, N1, N2, N3, and REM. Data included 3.5% of tied epochs. As a specialty, the scorers were able to mark their scoring as uncertain, if they were unsure about the sleep stage but still labelled with the most likely sleep stage, e.g. uncertain N1. Following this, 3936 epochs (10% of analysed data) were scored with uncertainty by at least one of the 10 scorers. The self‐applied PSG recordings were also used as completely separate test data in the validation analysis.

### 2.2 Automatic sleep staging

The presented aSAGA model utilises a fully convolutional neural network that has shown state‐of‐the‐art performance and good generalisability between different datasets in previous studies (Huttunen et al., 2022; Rusanen, Huttunen, et al., 2023). The architecture of the model was inspired by previous research conducted by Perslev et al. (Perslev et al., 2019; Perslev et al., 2021) and by Ronneberger et al. (Ronneberger et al., 2015). The model architecture used in aSAGA is the same as in previous studies and described in detail in Huttunen et al. (2022). The codes for setting up the model are available at https://github.com/rikuhuttunen/psg-simultscoring-models.

In the aSAGA, we used a single‐channel model which was first trained on EEG (C4‐M1) and then fine‐tuned with an EOG (E1‐M2) channel using the clinical PSG recordings. This was done to have generalisability between EEG and EOG channels and to increase the compliance of the model for frontal EEG and EOG channels of self‐applicable sensor setups. In the training, we utilised a recently published unified data format, called the *sleeplab format* (Huttunen et al., 2023), to have better reproducibility. The training of the model was conducted over 100 training passes. During each training epoch, each batch input to the model was created by sampling a 1 h segment of recording from eight different subjects. The start times of the segments were randomly sampled from each recording. Model weights were optimised using the AdamW algorithm (Loshchilov & Hutter, 2019). The categorical cross entropy was used as the loss function. Furthermore, instead of a constant learning rate, we used a learning rate scheduler (Smith, 2016). The learning rate division factor was 2.0 after every 20 epochs and the peak learning rate was set to 0.01. After initial training, a constant learning rate of 0.001 was used in the fine‐tuning. Otherwise, the finetuning was performed similarly to the initial training. The best model was returned after training according to validation accuracy. Training parameters and model parameters used in the training and finetuning are available in detail at https://github.com/matias-olavi/aSAGA.

All EEG and EOG signals are pre‐processed similarly to training data in the aSAGA, wherein they are bandpass filtered (0.3–32 Hz), re‐sampled to 64 Hz, and normalised with an interquartile range. The normalisation with an interquartile range was found to be effective in a previous study with cross‐domain predictions where peak‐to‐peak amplitude of the signal varied significantly (Rusanen, Huttunen, et al., 2023). Moreover, a previous study reported that EEG signals recorded in self‐applied PSGs have significantly lower peak‐to‐peak amplitudes compared with standard EEG derivations (Rusanen, Korkalainen, et al., 2023). Therefore, normalising each recording is a critical step in the preprocessing, although information about absolute amplitudes will be lost. Standard preprocessing functions of the *sleeplab format* extractor were used (available in GitHub). All signals were run through the single‐channel model to create an ensemble prediction of the hypnodensities i.e., the probability of each sleep stage for each 30 s epoch. Following this, the hypnodensities were averaged over the channels and the sleep stage with the highest probability was included in the final hypnogram.

### 2.3 Uncertainty mapping – the grey areas

Grey areas were derived from hypnodensities of manual and automatic sleep staging using uncertainty mapping. In the aSAGA, averaged hypnodensities from the ensemble predictions were used as the probability of the sleep stage in the studied epoch. In the manual sleep staging results obtained from 10 scorers, the probability for each sleep stage can be defined as the number of scorers of the overall 10 who scored the respective sleep stage. Let $p_{i}$, where *i* = 1, 2, ⋯,5 correspond to each of the five sleep stages, denote the probabilities or the hypnodensities for each 30 s epoch. We can estimate the uncertainty of the model from the hypnodensities with different metrics described in the literature (Monarch, 2021; Kang et al., 2021; Phan, Mikkelsen, et al., 2021). In the present paper, we compared the following five metrics:

1. Least confidence sampling
$$U_{L}=\left(1-\max\left(p_{I}\right)\right)*\frac{n}{n-1},$$

where $I=\left\{\mathrm{all}\;\text{sleep stages}\right\}$ and n is the number of sleep stages.

2 The margin of confidence sampling (between the two most confident labels)
$$U_{M}=1-\left(\max\left(p_{I}\right)-\max\left(p_{J}\right)\right),$$

where $I=\left\{\mathrm{all}\;\text{sleep stages}\right\}$, and J={allsleep stages exceptthe most probable}.

3 The ratio of confidence (between the two most confident labels)
$$U_{R}=\max\left(p_{I}\right)/\max\left(p_{J}\right),$$

where $I=\left\{\mathrm{all}\;\text{sleep stages}\right\}$, and J={allsleep stages exceptthe most probable}.

4 Coefficient of unalikeability
$$U_{U}=1-\sum_{i=1}^{n}{p_{i}}^{2}$$
5 Classification entropy
$$U_{E}=-\frac{\sum_{i=1}^{n}p_{i}\log_{2}p_{i}}{\log_{2}n},$$

where $0*\log_{2}\left(0\right)=0$ was assumed.

### 2.4 Validation analysis

To validate the single‐channel automatic sleep staging model, we first used the test set of clinical PSG recordings collected at the Princess Alexandra Hospital to show the sleep staging performance from the single EOG and EEG channels. Typical performance metrics such as accuracy, Cohen kappa, and F1‐scores were computed in an epoch‐wise manner to measure the overall sleep staging performance. Sleep stage‐specific classification performance was evaluated by computing precision, recall, and F1‐score for each class separately. Furthermore, we visualised the scorings between the automatic and manual sleep staging with a confusion matrix.

To validate the generalisability of the aSAGA, we evaluated the sleep staging performance with the Dreem Open Data. The performance of the automatic method was tested with healthy subjects and patients with OSA separately against the consensus of five scorers. In this validation, we utilised all EEG and EOG channels available in the dataset to create the ensemble prediction described in Section 2.3. Finally, the automatic sleep staging performance of the aSAGA was tested with the self‐applied PSGs against the consensus of the 10 scorers. No finetuning of the model was performed i.e., the performance was evaluated in a direct transfer manner with all datasets (sections 2.1.2 and 2.1.3).

Following the sleep staging performance validation, we tested the grey area concept of the aSAGA with two different analyses. First, we tested if excluding the most uncertain epochs of the prediction increased the automatic sleep staging performance when evaluated against the consensus manual scoring. This was done to evaluate if the grey areas corresponded to the areas of the recording where the confusion between manual and automatic sleep staging occurs. This analysis was performed using the uncertainty metrics proposed in Section 2.3 and varying the percentage of epochs considered as grey areas from 1% to 95% of the whole dataset based on the rank order of the uncertainty values. This validation was done using the Dreem Open Data, and the validation protocol is available at https://github.com/matias-olavi/aSAGA.

Second, we tested whether the grey areas of the aSAGA capture those areas of the self‐applied PSGs where the 10 scorers had the most confusion between each other. The testing was performed using the coefficient of unalikeability, which is suitable for capturing the variability of the manual scorings. A threshold of 0.6 was used for the uncertainty metric to separate grey areas from non‐grey areas with both the 10 scorers and the model hypnodensity series. Binary classification between the grey and non‐grey areas derived from manual and automatic sleep staging was computed. Confusions in the grey area classification between these two methods were visualised with a confusion matrix. Moreover, the results of this analysis were split into two parts, the epochs that were marked as uncertain by any of the 10 scorers, and the remaining data (not marked as uncertain by any of the scorers). The data consisting of epochs manually marked as uncertain represent grey areas that are known to exist in the sleep stage, whereas grey areas of data consisting of manually scored epochs that were not marked as uncertain represent more the uncertainties unknown for scorers.

## 3 RESULTS

### 3.1 Sleep staging performance

#### 3.1.1 Clinical PSG data–test set (*n* = 88)

**FIGURE 2:**
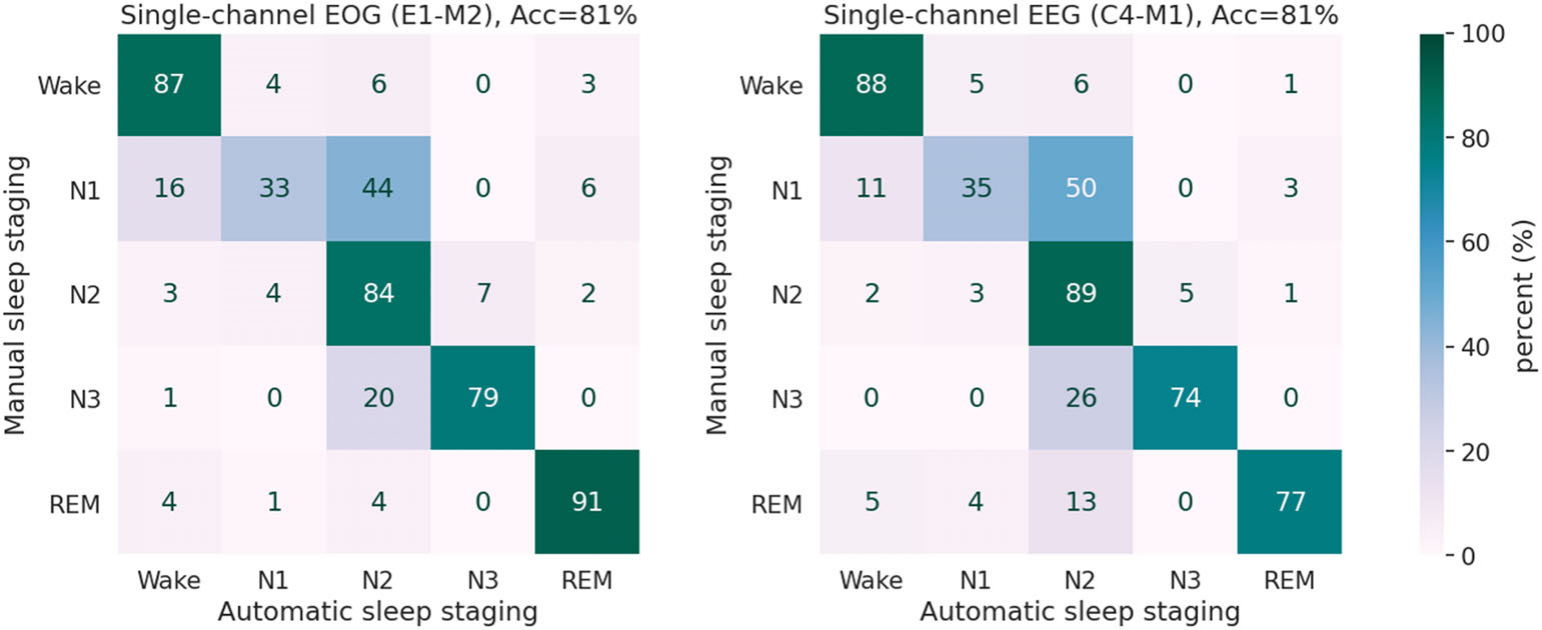
Confusion matrixes between manual sleep staging and the single‐channel outputs of automatic sleep staging with the aSAGA model. Performance was computed on the test set of the clinical polysomnographic recordings of suspected OSA patients (*n* = 88). Acc, Accuracy; aSAGA, Automatic Sleep Analysis with Grey Area; EEG, electroencephalography; EOG, electro‐oculography.

The single‐channel model performed well against manual sleep staging and with similar overall metrics (accuracy = 81% and κ = 0.74) for both EEG and EOG inputs (Table 1). Sleep stage‐specific metrics showed that REM sleep was better detected from the EOG input than from the EEG input, with F1‐scores of 0.88 and 0.83, respectively. In contrast, Wake was detected with similar consistency from EEG compared with EOG as an input, with F1‐scores of 0.90 and 0.89, respectively. Most of the confusions between automatic and manual sleep staging occurred in the classification of non‐REM sleep stages of N1, N2, and N3 (Figure 2).

**TABLE 1:** Performance metrics of the automatic sleep staging model in a test set of the clinical PSG data when single‐channel inputs were used.

Recording types	Subjects	Signal	Sleep stage	Performance metrics			Number of epochs
				Precision	Recall	F1‐score	
Type I PSG	Suspected OSA (*n* = 88)	EOG (E1‐M2)	Wake	0.91	0.87	0.89	26,881
			N1	0.51	0.33	0.40	6708
			N2	0.75	0.84	0.80	25,370
			N3	0.80	0.79	0.80	9,695
			REM	0.85	0.91	0.88	9567
			Macro average	0.76	0.75	0.75	78,221
			κ	0.74			78,221
			Accuracy	81%			78,221
		EEG (C4‐M1)	Wake	0.93	0.88	0.90	26,881
			N1	0.50	0.35	0.41	6708
			N2	0.72	0.89	0.80	25,370
			N3	0.85	0.74	0.79	9695
			REM	0.91	0.77	0.83	9567
			Macro average	0.78	0.73	0.75	78,221
			κ	0.74			78,221
			Accuracy	81%			78,221

*Note*: Metrics were computed against the manual sleep staging. The macro average is the unweighted average over the sleep stage‐specific metrics.

Abbreviations: epoch, 30‐second signal segment; κ, Cohen's kappa; EEG, electroencephalography; EOG, electro‐oculography; OSA, obstructive sleep apnea; PSG, polysomnography; REM, rapid eye movement.

#### 3.1.2 Dreem open data (*n* = 25 + 56)

The aSAGA model showed high generalisability of the sleep staging performance to separate open‐access datasets consisting of recordings of healthy subjects (DOD‐H, *n* = 25) and subjects with OSA (DOD‐O, *n* = 56). With DOD‐H data, the aSAGA model reached 80% accuracy, and with DOD‐O data the accuracy was 82% without any transfer learning approaches. Sleep stages Wake, N2, and REM were detected with a good agreement (F1‐scores ≥0.82) against the majority scoring (Table 2). Slightly lower performance was reached for the detection of N3 sleep, with F1‐scores being 0.78 and 0.79 for DOD‐H and DOD‐O data, respectively. However, N1 sleep was detected with moderate agreement in both datasets.

**TABLE 2:** Performance metrics for the automatic sleep staging in Dreem Open Data when the aSAGA model was used.

Recording type	Subjects	Signals	Sleep stage	Performance metrics			Number of epochs
				Precision	Recall	F1‐score	
Type I PSG	Healthy (*n* = 25)	All EEG + EOG channels	Wake	0.87	0.77	0.82	3037
			N1	0.71	0.29	0.41	1505
			N2	0.83	0.83	0.83	11,879
			N3	0.65	0.97	0.78	3514
			REM	0.87	0.78	0.82	4727
			Macroaverage	0.79	0.73	0.73	24,662
			κ	0.71			24,662
			Accuracy	80%			24,662
	OSA (*n* = 56)		Wake	0.90	0.79	0.84	10,660
			N1	0.50	0.42	0.46	2898
			N2	0.85	0.86	0.86	26,650
			N3	0.71	0.89	0.79	5683
			REM	0.85	0.82	0.83	8306
			Macro average	0.76	0.76	0.76	54,197
			κ	0.74			54,197
			Accuracy	82%			54,197

*Note*: Metrics were computed against the majority scores of manual sleep staging. Macro average is the unweighted average over the sleep stage‐specific metrics.

Abbreviations: aSAGA, Automatic Sleep Analysis with Grey Areas; epoch, 30‐second signal segment; κ, Cohen's kappa; EEG, electroencephalography; EOG, electro‐oculography; OSA, obstructive sleep apnea; PSG, polysomnography; REM, rapid eye movement.

#### 3.1.3 Self‐applied PSGs (*n* = 50)

**FIGURE 3:**
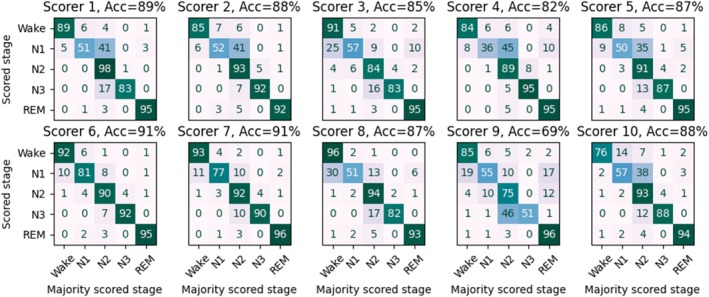
Confusions between the 10 individual scorings and the majority score derived from manual sleep staging in self‐applied polysomnographic recordings (*n* = 50). Epochs manually marked as uncertain were excluded from the analysis. Acc, accuracy; REM, rapid eye movement.

**FIGURE 4:**
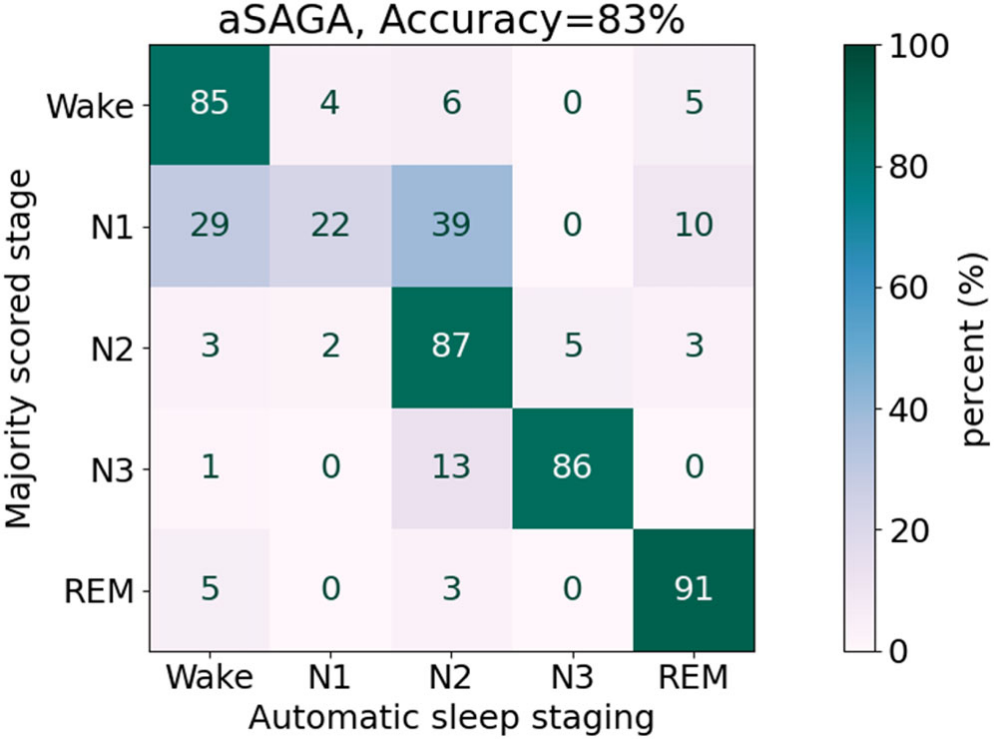
Confusions between automatic sleep staging of aSAGA model and the majority score derived from manual sleep staging in self‐applied home sleep recordings. Epochs manually marked as uncertain were excluded from the analysis. aSAGA, Automatic Sleep Analysis with Grey Areas; REM, rapid eye movement.

Manual sleep staging of the self‐applied PSGs showed high variation in the agreement of the 10 individual scorings against the majority score with accuracy ranging from 69% to 91% between the 10 scorers (Figure 3). On bar with these manual scorings against the majority score, the aSAGA reached the accuracy of 83% against the majority scoring. High F1‐scores (≥0.86) were reached for detecting N2, N3, and REM sleep (Table 3, Figure 4). The F1‐score for detecting Wake was lower, however. This was due to a precision of 0.63, although a high recall of 0.85 was reached. Similarly, as in the manual scorings, stage N1 was detected with only a fair agreement (F1‐score = 0.30), and mostly confused with Wake and N2 stages (Figure 4).

**TABLE 3:** Performance metrics for the automatic sleep staging in self‐applied polysomnographic (PSG) recordings when the aSAGA model was used.

Recording type	Subjects	Signals	Sleep stage	Performance metrics			Number of epochs
				Precision	Recall	F1‐score	
Self‐applied PSG	Healthy to severe OSA (*n* = 50)	All EEG + EOG channels	Wake	0.63	0.85	0.73	3183
			N1	0.50	0.22	0.30	2303
			N2	0.88	0.87	0.87	17,443
			N3	0.87	0.86	0.86	6136
			REM	0.85	0.91	0.88	6168
			Macro average	0.75	0.74	0.73	35,233
			κ	0.75			35,233
			Accuracy	83%			35,233

Metrics were computed against the majority scores of manual sleep staging. Epochs manually scored with uncertainty were excluded from the analysis. Macro average is the unweighted average over the sleep stage‐specific metrics.

Abbreviations: aSAGA, Automatic Sleep Analysis with Grey Areas; EEG, electroencephalography; EOG, electro‐oculography; epoch, 30‐second signal segment; κ, Cohen's kappa; SAS, Self‐Applied Somnography (Nox Medical).

### 3.2 Grey area validity

#### 3.2.1 Increase in sleep staging performance

**FIGURE 5:**
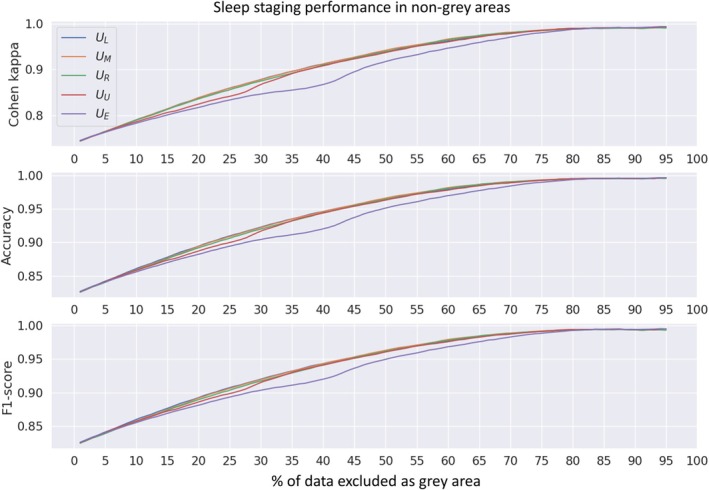
Performance metrics of the aSAGA model in automatic sleep staging as a function of the amount of data excluded as grey areas. Grey areas were defined based on different hypnodensity‐based uncertainty metrics (U) to compare the effectiveness of these measures. The compared metrics were the least confidence $U_{L}$, the margin of confidence $U_{M}$, the ratio of confidence $U_{R}$, the coefficient of unalikeability $U_{U}$, and the classification entropy $U_{E}$. F1‐score is the weighted average of class‐wise F1‐scores. Dreem Open Data with obstructive sleep apnea subjects was used in the analysis (*n* = 56 full‐night polysomnographies). aSAGA, Automatic Sleep Analysis with Grey Areas.

Automatic sleep staging performance increased effectively as a function of the amount of excluded data i.e., the grey areas, based on all of the compared uncertainty metrics in the DOD‐O data (Figure 5). Differences in increased sleep staging performance were negligible between the different uncertainty metrics on less than 10% of excluded data (Figure 5). However, classification entropy (*U*_*E*_) showed less increase in sleep staging performance between 10% and 75% of excluded data, than the other metrics. Similarly, the coefficient of unalikeability (*U*_*U*_) increased the sleep staging performance slightly less between 10% and 30% of excluded data than the more direct measures of hypnodensities (*U*_*L*_, *U*_*M*_, and *U*_*R*_).

**FIGURE 6:**
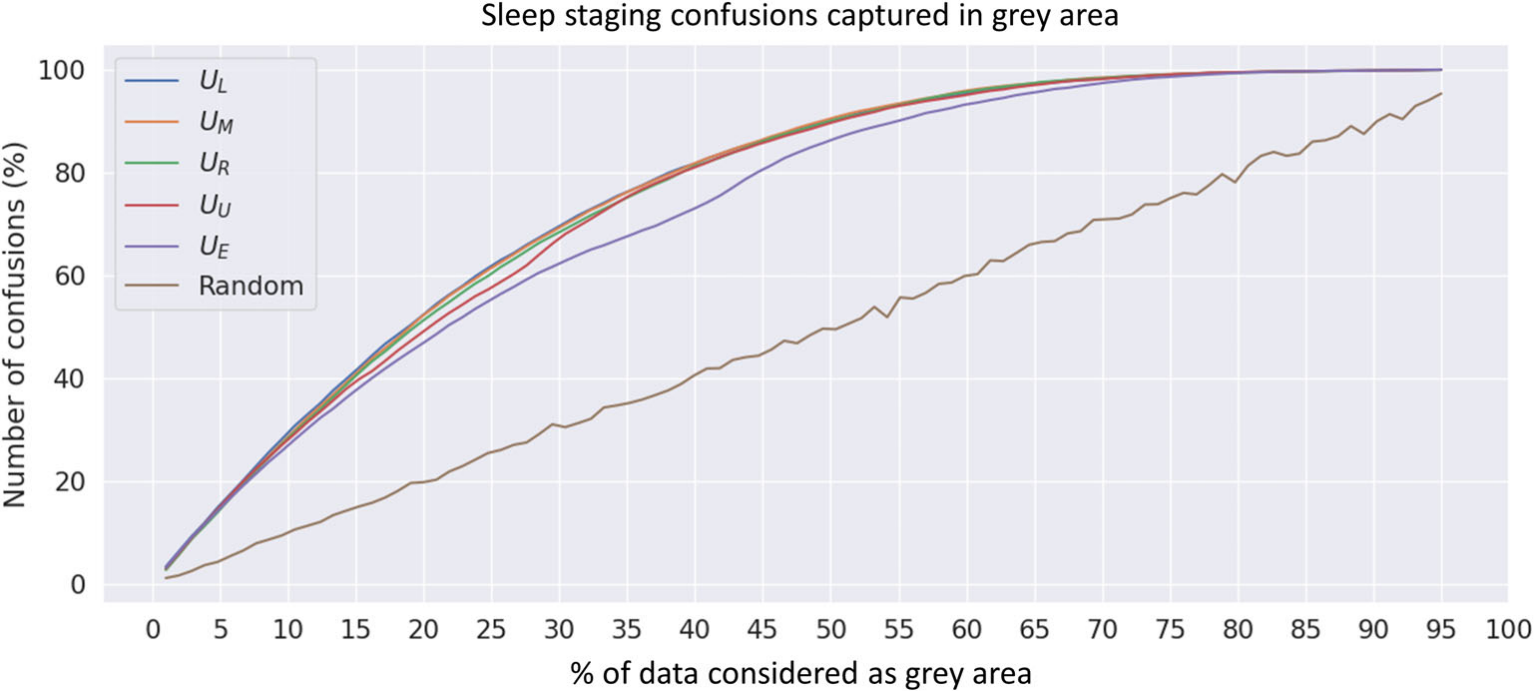
Percentage of sleep staging confusion between automatic and manual majority scoring captured in the model grey areas. Grey areas were defined based on different hypnodensity‐based uncertainty metrics (U) to compare the effectiveness of these measures. The compared metrics were the least confidence $U_{L}$, the margin of confidence $U_{M}$, the ratio of confidence $U_{R}$, the coefficient of unalikeability $U_{U}$, and the classification entropy $U_{E}$. Dreem Open Data with obstructive sleep apnea subjects was used in the analysis (n = 56 full‐night polysomnographies). aSAGA, Automatic Sleep Analysis with Grey Areas.

Furthermore, confusions in sleep staging between manual and automatic scoring were captured effectively into grey areas (Figure 6). For example, when considering 40% of the data as grey areas, over 80% of the confusion between manual and automatic sleep staging was captured with *U*_*L*_, *U*_*M*_, *U*_*R*_, and *U*_*U*_ metrics. However, entropy‐based metrics *U*_*U*_ and *U*_*E*_ showed slightly worse overall performance than the more direct measures of hypnodensities *U*_*L*_, *U*_*M*_, and *U*_*R*_.

#### 3.2.2 Agreement with manual scoring confusions in self‐applied PSG

**FIGURE 7:**
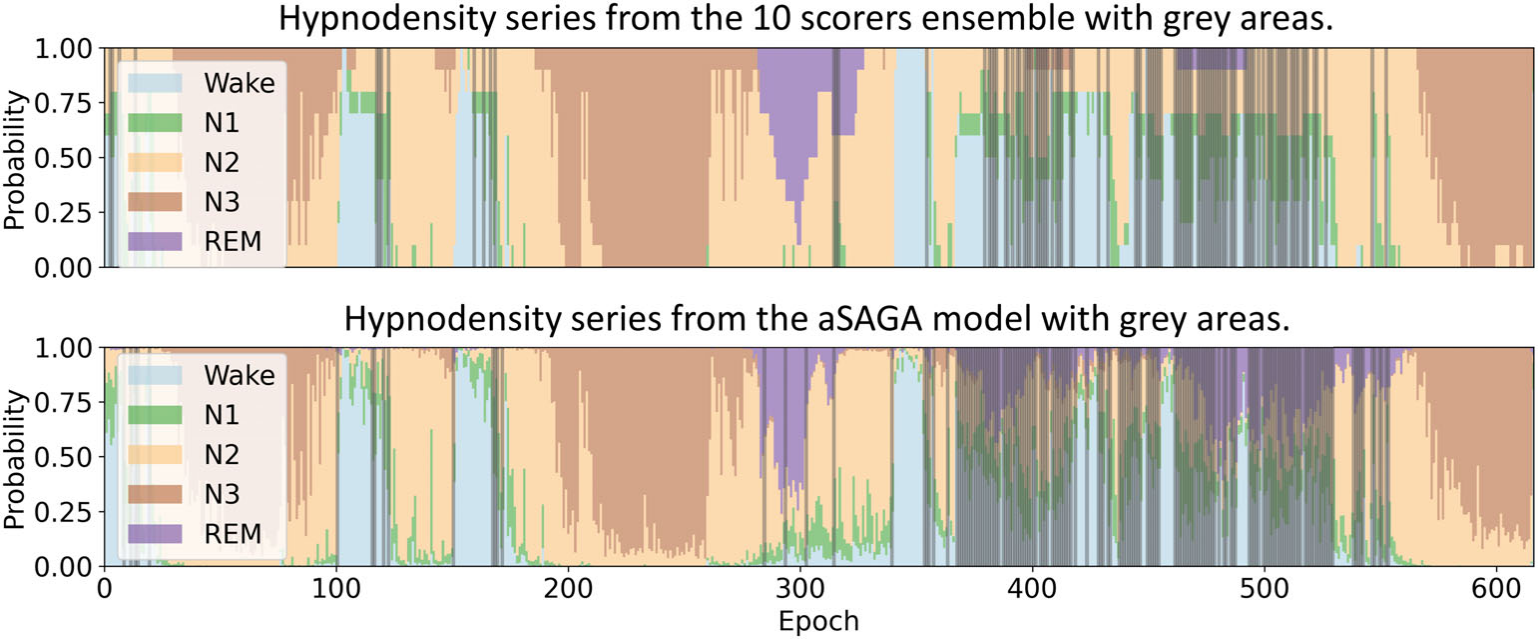
An example of hypnodensity series and grey areas derived from manual sleep staging confusions and aSAGA model outputs for one of the self‐applied home sleep recordings. Hypnodensities are shown as stacked bars with the respective colours indicated in the legend. Grey areas are shown as shaded vertical bars of a grey colour corresponding to epochs where the coefficient of unalikeability was higher than 0.6. aSAGA, Automatic Sleep Analysis with Grey Areas.

An example of the hypnodensity series and grey areas derived from manual and automatic sleep staging of one recording is shown in Figure 7. Defining the grey areas similarly from the 10 scorers and the automatic model's hypnodensity (with threshold *U*_*U*_ >0.6) series resulted in a median of 2.4% and 10.3% of analysed epochs as grey areas in the 50 recordings, respectively.

**FIGURE 8:**
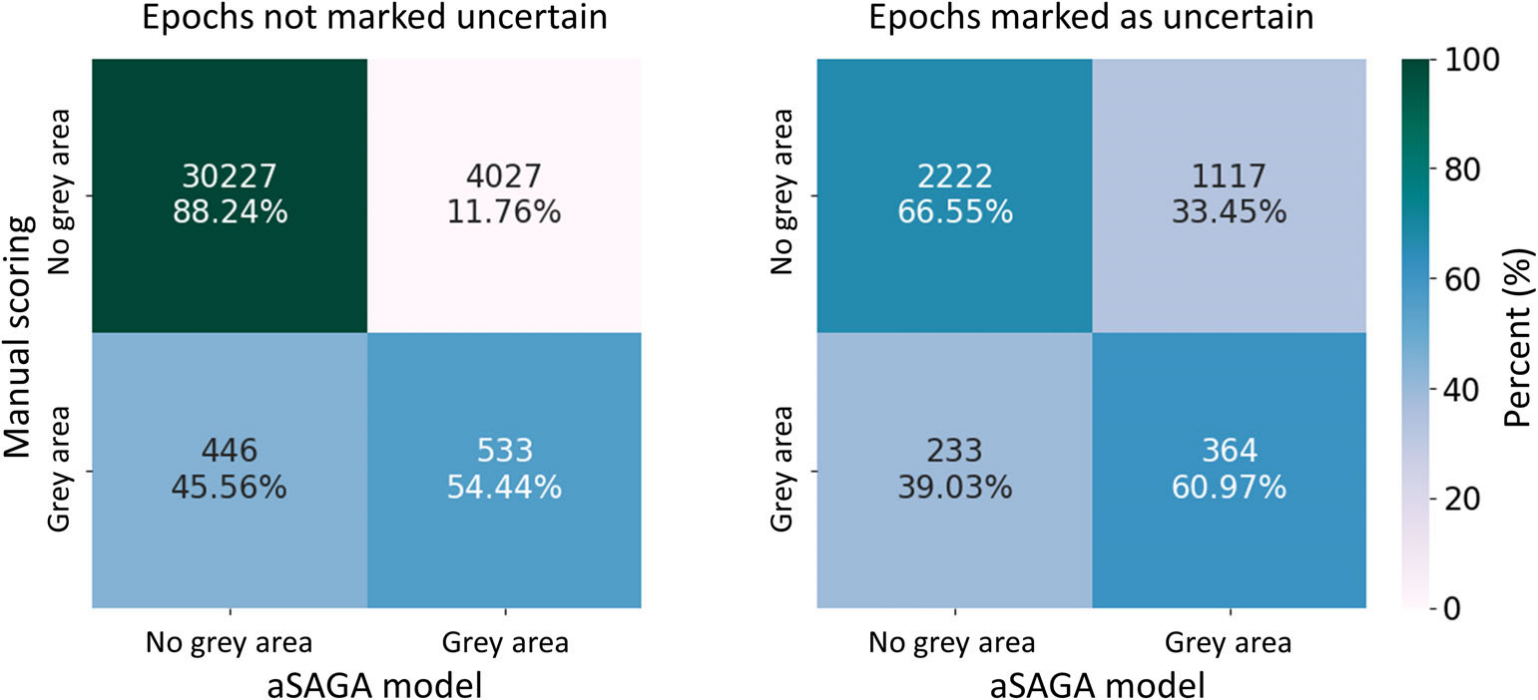
Confusions in the grey areas derived from 10 manual scorings and the aSAGA model output. The left confusion matrix represents those areas of the recording that were not annotated as uncertain by any of the 10 scorers while scoring the sleep stages. The right confusion matrix is for epochs that were marked as uncertainty at least by one of the 10 scorers while still scoring the most likely sleep stage (10% of all data). aSAGA = Automatic Sleep Analysis with Grey Areas.

When all epochs were split into marked uncertainties and unmarked uncertainties, the model grey areas agreed better with the manual scoring grey areas within the marked uncertainties than within the unmarked uncertainties (Figure 8). In both cases, the model captured most of the manual scoring grey areas, i.e., 54% within unmarked uncertainties and 61% within the marked uncertainties.

## 4 DISCUSSION

This paper presents a novel human‐in‐the‐loop concept for sleep staging that incorporates the integration of automatic analysis and manual review of the automatic output. The efficacy of various hypnodensity‐based uncertainty metrics was evaluated in delineating the grey areas within automatic sleep staging and these grey areas compared with those observed in manual sleep staging. Through preclinical validation, we observed that the performance of the automatic sleep staging model was comparable to that of the manual scorers in different recording types. Hypnodensity‐based metrics of uncertainty show potential in defining the grey areas that capture confusions in automatic versus manual sleep staging. Additionally, the grey areas identified from the automatic sleep staging captured most of the regions of the recording where significant discrepancies between multiple manual scorers occurred.

Consequently, focussing on the manual revision of the grey areas alone could enhance the accuracy of sleep staging, while simultaneously minimising the workload associated with fully manual sleep staging. By leveraging the concept of grey areas, the proposed approach offers a valuable user‐assistive solution for improving the reliability of sleep staging, especially with unconventional sensor setups. At the same time, transparency of uncertainty can reduce the scepticism related to the application of machine learning methods in clinical medicine (Linardatos et al., 2021; Rudin, 2019), which is one of the greatest challenges in implementing automatic PSG scoring at scale (Alvarez‐estevez, 2023). Nevertheless, further research is warranted to evaluate the clinical feasibility, incorporate active learning (Monarch, 2021), and to quantify the reduction in working hours achievable with this approach.

A few previous studies have investigated the uncertainty of automatic sleep staging, utilising various approaches that should be discussed (Bakker et al., 2022; Fiorillo et al., 2021; Hong et al., 2021; Kang et al., 2021; Phan, Mikkelsen, et al., 2021). Phan et al. focussed on a single entropy metric for hypnodensities and employed a fixed threshold to assess sleep staging accuracy in uncertain and certain epochs (Phan, Mikkelsen, et al., 2021). Kang et al. conducted a study involving the use of model uncertainty to enhance sleep staging accuracy, examining 20 recordings (Kang et al., 2021). However, their findings revealed statistically significant improvement in Cohen's kappa only for REM vs. N3, rather than for agreement across all five stages. Fiorillo et al. and Hong et al. introduced sophisticated models capable of estimating the uncertainty of automatic sleep staging, aiming to capture discrepancies between manual and automatic approaches (Fiorillo et al., 2021; Hong et al., 2021). Previous validation analyses have primarily focussed on evaluating the extent of discrepancies between manual and automatic scoring within uncertain epochs, without considering the true grey areas associated with manual sleep staging. Only one study compared hypnodensities derived from multiple manual scorings against automatic scoring, revealing a strong correlation between the two (Bakker et al., 2022). However, none of these works have demonstrated the generalisability of their methods across different recording types. In this paper, we contribute to the field by considering both validation aspects and demonstrating the generalisability of our approach across diverse recording types. Additionally, as a novel contribution, we compare different uncertainty metrics and aim to stimulate discussion surrounding various strategies for defining the grey areas of automatic sleep analysis.

In this study, we defined grey areas using two different approaches. Firstly, when comparing uncertainty metrics, grey areas were defined as a percentage of the most uncertain epochs within the recording. Secondly, when comparing uncertainties between the automatic and multiple manual scorings in self‐applied PSGs, grey areas were determined using a fixed threshold for the applied uncertainty metric.

Using the threshold method, the number of grey areas varied across recordings, reflecting the inherent variations in the difficulty of scoring different recordings, e.g., healthy vs. OSA subjects (Boostani et al., 2017). It is natural for some recordings to present greater challenges in analysis than others, and theoretically, the threshold method should capture this. However, when employing a fixed value for uncertainty, the calibration of the model influences the number of grey areas. If the model is applied to data differing from the training data, higher uncertainties may be obtained, resulting in an increased number of grey areas. Now the model was trained on standard PSG signals and applied on the forehead EEG of the self‐applied PSGs. This could explain why the threshold method yielded more grey areas based on model hypnodensities compared with 10 scorer hypnodensities in self‐applied PSGs (with a median percentage of 2.4% for manual scoring and 10.3% for the model).

Conversely, a fixed percentage of the most uncertain epochs as grey areas could be considered, and then the number of grey areas would remain constant for each recording. However, this approach also has its limitations. Recordings that pose greater challenges for the model's analysis would be treated the same as recordings that are easier to analyse. Consequently, some true grey areas might be overlooked in difficult‐to‐analyse recordings, or already correctly analysed epochs might be highlighted in easier‐to‐analyse recordings, which would not be a good use of the manual scorer's time.

This study was limited to comparing the grey areas derived from hypnodensities averaged over predictions from multiple EEG and EOG channels. Although this averaged ensemble prediction appears suitable for generalising over different recording types with various EEG and EOG derivations, some information about the source of the uncertainty might be lost. This information may differentiate the uncertainty associated with data quality from the uncertainty emerging from manual scoring discrepancies in the training data, although they are likely not fully distinct. These two sources of uncertainty are not currently separated by the proposed model.

The most effective method to define the grey areas for the interaction between manual and automatic sleep staging remains uncertain. Ideally, the sensitivity for grey areas, such as the threshold for uncertainties or the percentage of most uncertain epochs, could be interactively adjusted for each recording, based on the expertise of the sleep technologist. This personalised approach could optimise the identification of grey areas and enhance the overall performance of the sleep staging process. Another approach would be to use, for example, automatic domain adaptation methods and to calibrate the model for each recording type separately (Phan, Chen, et al., 2021). Furthermore, model uncertainty is an active area of machine learning research, and novel ways to quantify it, such as Monte Carlo dropout, should be tested further in the field of sleep medicine (Monarch, 2021; Abdar et al., 2021; Fiorillo et al., 2021).

## 5 CONCLUSION

Through extensive evaluation and comparison, we have herein demonstrated that the proposed automatic sleep staging model, along with the identification of grey areas, can achieve similar performance to manual scorers on various types of sleep recordings. Different hypnodensity‐based uncertainty metrics are shown to effectively capture areas of sleep recordings that should be re‐analysed for increased sleep staging accuracy. A significant portion of the automatically identified grey areas overlap with those areas of the recordings where confusions between expert‐based scorings appear. The two approaches employed for defining grey areas, namely the percentage‐based method and the fixed threshold method, offer distinct advantages and trade‐offs. However, the optimal approach for determining grey areas should be personalised, considering the expertise of sleep technologists and the specific characteristics of each recording. In conclusion, this study introduces a way to combine manual and automatic sleep staging and provides an investigation of the uncertainty in automatic sleep staging that brings us closer to integrating human‐in‐the‐loop scoring into clinical workflows.

## FUNDING INFORMATION

This work was supported by the European Union's Horizon 2020 research and innovation programme under grant agreement No 965417; by the State Research Funding for university‐level health research, Kuopio University Hospital, Wellbeing service county of North Savo (Grants 5041807, 5041789, 5041794, 5041797, 5041803, 5041809, 5041804); by the Finnish Cultural Foundation through Kainuu Regional Fund and Central Fund; by the Emil Aaltonen Foundation; by the Research Foundation of the Pulmonary Diseases, by the Foundation of the Finnish Anti‐Tuberculosis Association; by Tampere Tuberculosis Foundation; by the NordForsk (NordSleep Project 90458) through Business Finland (Grant 5133/31/2018); by the French National Research Agency, framework of the Investissements MIAI Artificial Intelligence chairs of excellence from the Grenoble Alpes (grants ANR‐15‐IDEX‐02 and ANR‐19‐P3IA‐0003) and the Icelandic Research Fund.

## CONFLICT OF INTEREST STATEMENT

ESA reports honoraria from ResMed, Nox Medical, Jazz Pharmaceuticals, Linde Healthcare, Alcoa–Fjardaral, Wink Sleep, Apnimed, and Vistor. She is also a member of the Philips Medical Advisory Board. Other authors declare no conflict of interest relevant to this study.

## Supporting information


**DATA S1** Supporting Information.

## Data Availability

The private clinical data from Princess Alexandra Hospital include sensitive medical information and can therefore not be made publicly available. The self‐applied PSGs are stored in a safe medical data environment for research purposes, but not made publicly available. Dreem Open Data is publicly available.
