# Supplementary material for: Retrospective validation of automatic sleep analysis with grey areas model for human‐in‐the‐loop scoring approach
Source: J Sleep Res. 2024 Oct 23;34(3):e14362. doi: 10.1111/jsr.14362 (PMC12069739; doi:10.1111/jsr.14362)
Supplement: Supplementary file 1 — DATA S1 Supporting Information. [file JSR-34-e14362-s001.docx]

# **Supplementary material**

| **Table S1**. Demographic information of test dataset subjects (*n*=88) in clinical PSG recordings data | | | | | | | | | | |
| --- | --- | --- | --- | --- | --- | --- | --- | --- | --- | --- |
|  | Sex | Age [years] | BMI [kg/m^2] | TST [minutes] | WASO [minutes] | N1% | N2% | N3% | REM% | AHI [1/h] |
| males | 51% |  |  |  |  |  |  |  |  |  |
| mean |  | 55.62 | 34.57 | 291.70 | 120.74 | 13.71 | 49.11 | 19.48 | 17.70 | 24.35 |
| std |  | 13.15 | 7.97 | 86.30 | 64.21 | 10.62 | 11.93 | 14.35 | 7.29 | 28.16 |
| min |  | 23 | 17.20 | 82.00 | 23.00 | 1.30 | 11.20 | 0.00 | 0.00 | 0.20 |
| 25% |  | 45 | 29.63 | 230.50 | 64.88 | 6.08 | 42.80 | 8.20 | 13.33 | 7.85 |
| 50% |  | 56 | 33.65 | 298.75 | 116.50 | 12.35 | 49.50 | 18.85 | 18.60 | 15.00 |
| 75% |  | 65 | 38.48 | 354.75 | 158.00 | 17.65 | 57.30 | 25.80 | 21.53 | 30.65 |
| max |  | 85 | 66.20 | 480.50 | 326.50 | 68.40 | 83.00 | 72.00 | 38.80 | 144.80 |
| BMI = Body Mass Index, N1-N3% = Percent of Stage N1-N3 sleep, TST = Total Sleep Time, REM% = Percent of Stage REM sleep, WASO = Wake After Sleep Onset, AHI = Apnea Hypopnea Index (events/hours of sleep). | | | | | | | | | | |

| **Table S2.** Demographic information of validation dataset subjects (*n*=78) in clinical PSG recordings data. | | | | | | | | | | |
| --- | --- | --- | --- | --- | --- | --- | --- | --- | --- | --- |
|  | Sex | Age [years] | BMI [kg/m^2] | TST [minutes] | WASO [minutes] | N1% | N2% | N3% | REM% | AHI [1/h] |
| males | 65% |  |  |  |  |  |  |  |  |  |
| mean |  | 54.11 | 34.50 | 309.36 | 107.28 | 15.90 | 50.24 | 17.55 | 16.50 | 27.84 |
| std |  | 14.74 | 8.78 | 84.96 | 57.66 | 12.40 | 12.02 | 11.99 | 8.54 | 27.36 |
| min |  | 20 | 21.70 | 89.00 | 13.00 | 1.50 | 20.70 | 0.00 | 0.00 | 0.20 |
| 25% |  | 43 | 27.95 | 253.00 | 61.88 | 7.70 | 43.13 | 9.40 | 11.20 | 6.05 |
| 50% |  | 54 | 33.10 | 312.75 | 101.25 | 12.55 | 50.35 | 17.70 | 16.60 | 19.80 |
| 75% |  | 65 | 38.68 | 362.13 | 150.38 | 22.15 | 56.50 | 24.30 | 23.10 | 44.40 |
| max |  | 84 | 64.50 | 486.00 | 287.00 | 79.30 | 89.20 | 58.50 | 38.50 | 116.00 |
| One subject did not want to report their sex and one subject had missing demographic data. BMI = Body Mass Index, N1-N3% = Percent of Stage N1-N3 sleep, TST = Total Sleep Time, REM% = Percent of Stage REM sleep, WASO = Wake After Sleep Onset, AHI = Apnea Hypopnea Index (events/hours of sleep). | | | | | | | | | | |

| **Table S3.** Demographic information of training dataset subjects (*n*=710) in clinical PSG recordings data. | | | | | | | | | | |
| --- | --- | --- | --- | --- | --- | --- | --- | --- | --- | --- |
|  | Sex | Age | BMI [kg/m^2] | TST [minutes] | WASO [minutes] | N1% | N2% | N3% | REM% | AHI [1/h] |
| males | 54% |  |  |  |  |  |  |  |  |  |
| mean |  | 54.21 | 35.84 | 303.06 | 112.12 | 14.93 | 48.16 | 19.79 | 17.12 | 24.13 |
| std |  | 14.60 | 9.99 | 79.50 | 66.18 | 12.77 | 12.41 | 13.64 | 8.14 | 23.59 |
| min |  | 17 | 0.00 | 67.50 | 7.50 | 0.00 | 5.50 | 0.00 | 0.00 | 0.00 |
| 25% |  | 44 | 29.30 | 257.63 | 61.00 | 6.63 | 41.13 | 9.98 | 11.93 | 7.10 |
| 50% |  | 55 | 34.70 | 309.00 | 101.25 | 10.80 | 47.90 | 18.50 | 17.15 | 15.60 |
| 75% |  | 65 | 40.65 | 360.00 | 148.38 | 18.78 | 55.90 | 27.48 | 22.20 | 32.30 |
| max |  | 88 | 76.20 | 531.50 | 407.00 | 87.10 | 87.20 | 89.30 | 47.10 | 143.10 |
| BMI = Body Mass Index, N1-N3% = Percent of Stage N1-N3 sleep, TST = Total Sleep Time, REM% = Percent of Stage REM sleep, WASO = Wake After Sleep Onset, AHI = Apnea Hypopnea Index (events/hours of sleep). | | | | | | | | | | |

| **Table S4.** Demographic information of subjects (*n*=48) in self-applied PSG recordings data. | | | | | | | | | | |
| --- | --- | --- | --- | --- | --- | --- | --- | --- | --- | --- |
|  | Sex | Age | BMI [kg/m^2] | TST [minutes] | WASO [minutes] | N1% | N2% | N3% | REM% |  |
| males | 46% |  |  |  |  |  |  |  |  |  |
| mean |  | 48.82 | 28.14 | 391.76 | 37.12 | 8.18 | 53.79 | 19.30 | 19.49 |  |
| std |  | 17.21 | 6.26 | 79.37 | 37.50 | 7.14 | 8.97 | 9.69 | 7.36 |  |
| min |  | 22 | 19.61 | 192.50 | 2.50 | 1.43 | 33.40 | 1.55 | 0.94 |  |
| 25% |  | 32 | 29.30 | 347.00 | 13.75 | 4.20 | 48.46 | 12.23 | 15.82 |  |
| 50% |  | 50 | 26.87 | 404.00 | 24.00 | 5.75 | 52.37 | 18.25 | 20.16 |  |
| 75% |  | 61 | 31.45 | 444.12 | 45.62 | 9.99 | 60.38 | 25.20 | 23.58 |  |
| max |  | 81 | 50.73 | 540.50 | 192.50 | 35.61 | 72.52 | 42.72 | 37.94 |  |
| Two subjects had missing demographic data. Sleep stage percentages are computed from the majority sleep stage sequence. BMI = Body Mass Index, N1-N3% = Percent of Stage N1-N3 sleep, TST = Total Sleep Time, REM% = Percent of Stage REM sleep, WASO = Wake After Sleep Onset, AHI = Apnea Hypopnea Index (events/hours of sleep). | | | | | | | | | | |
